# Survival outcomes and prognostic factors for first-line abiraterone acetate or enzalutamide in patients with metastatic castration-resistant prostate cancer

**DOI:** 10.1186/s12885-023-10885-4

**Published:** 2023-06-20

**Authors:** Chi-Shin Tseng, Jui-Han Yang, Shi-Wei Huang, Yu-Jen Wang, Chung-Hsin Chen, Yeong-Shiau Pu, Jason Chia-Hsien Cheng, Chao-Yuan Huang

**Affiliations:** 1 Graduate Institute of Clinical Medicine, National Taiwan University College of Medicine, No.1, Jen Ai road, Section 1, Taipei, Taiwan; 2 Department of Urology, National Taiwan University Hospital, National Taiwan University, Taipei, Taiwan; 3 Department of Education, National Taiwan University Hospital, Taipei, Taiwan; 4 Department of Urology, National Taiwan University Hospital Yun-Lin Branch, Taipei, Taiwan; 5 Division of Radiation Oncology, Department of Oncology, National Taiwan University Hospital and National Taiwan University College of Medicine, Taipei, Taiwan; 6 Department of Radiation Oncology, School of Medicine, Fu-Jen Catholic University Hospital and College of Medicine, New Taipei City, Taiwan

**Keywords:** Prostatic neoplasms, Abiraterone acetate, Enzalutamide, Androgens, Castration

## Abstract

**Purpose:**

To investigate the survival outcomes of metastatic castration-resistant prostate cancer (mCRPC) patients receiving first-line novel androgen receptor axis-targeted therapies (ARATs) and prognostic factors for patient survival.

**Methods:**

This retrospective study obtained data from 202 patients who started abiraterone acetate or enzalutamide as first-line therapy for mCRPC between 2016 and 2021 from a single academic center. The primary endpoint was overall survival (OS) defined as the interval from the start of ARAT to death, loss to follow-up, or the end of the study period. The secondary endpoints were PSA decline, PSA nadir, and time to nadir (TTN) after ARATs. Kaplan–Meier survival analyses were applied for depicting OS. Cox proportional hazards model with inversed probability of treatment weighing-adjustment was used to validate the effect of patient, disease, and treatment response factors on OS.

**Results:**

Among 202 patients, 164 patients were treated with first-line ARATs alone and 38 patients received second-line chemotherapy. The median OS was not reached in patients with first-line ARATs alone and was 38.8 months in those with subsequent chemotherapy after failure from ARATs. OS was not different between the use of abiraterone and enzalutamide, though enzalutamide showed a higher rate of PSA decline ≧ 90% (56% versus 40%, p = 0.021) and longer TTN (5.5 versus 4.7 months, p = 0.019). Multivariable analysis showed that PSA nadir > 2 ng/mL [hazard ratio (HR) 7.04, p < 0.001] and TTN<7 months (HR 2.18, p = 0.012) were independently associated with shorter OS. Patients with both of these poor prognostic factors had worse OS compared to those who had 0–1 factors (HR 9.21, p < 0.001).

**Conclusions:**

Patients with mCRPC who received first-line ARATs had better survival if they had a PSA nadir$\leqq$2 ng/mL or a TTN$\geqq$7 months. Further study is needed to determine if an early switch in therapy for those in whom neither is achieved may impact OS.

**Supplementary Information:**

The online version contains supplementary material available at 10.1186/s12885-023-10885-4.

## Introduction

Androgen deprivation therapy (ADT) has been the fundamental strategy for the treatment of metastatic prostate cancer. However, almost all patients acquired resistance to ADT after years of treatment, known as castration-resistant prostate cancer (CRPC). Although patients with metastatic CRPC (mCRPC) are in the terminal stage of the disease, their median overall survival (OS) in real world data can reach 19–30 months under the continuous progress of treatment modalities [1–3]. A new generation of androgen receptor axis-targeted therapies (ARATs), abiraterone acetate and enzalutamide, has been shown to be effective in prolonging the lifespan of patients with mCRPC as first-line treatment [4–6]. ARATs are administered orally to block the androgen pathway, including abiraterone acetate, a CYP 17 inhibitor blocking androgen synthesis [7], and enzalutamide, an androgen receptor antagonist [8]. There were many meta-analysis studies analyzing the outcomes of these two drugs for first-line use, but the results were not consistent. Two meta-analyses for clinical trials including PREVAIL and COU-AA-302 suggested that enzalutamide has better survival than abiraterone acetate [9, 10]. However, a network meta-analysis found no difference in survival between the two drugs [11].

On the other hand, as treatment methods change and advance, new clinical data need to be studied in order to understand which factors are effective in predicting treatment outcomes and patient survival. The use of nadir prostate-specific antigen (PSA) level and time to nadir (TTN) as prognostic factors for metastatic hormone-sensitive prostate cancer after ADT treatment has been reported in the literature [12–15]. In addition, nadir PSA and TTN in response to initial ADT can also predict survival in mCRPC patients [16]. However, no study has reported whether the nadir PSA and TTN after first-line ARATs in patients with mCRPC can predict patient survival. Therefore, this study will discuss survival outcomes in patients with mCRPC treated with first-line ARATs, abiraterone and enzalutamide, and explore factors associated with OS.

## Materials and methods

The Institutional Review Board of National Taiwan University Hospital approved this study. Informed consent was waived for the study due to minimal risk to the subjects.

### Study population and treatment details

We retrospectively collected clinical data and electronic medical records of all patients treated with abiraterone acetate or enzalutamide between July 1, 2016 and June 31, 2021 in a single academic center. During this study period, we identified patients with mCRPC who received ARAT as first-line therapy. All ARAT drugs were only issued and reimbursed after applying to the National Health Insurance Administration by reporting pathology report, the record of the use of ADT, image, serial PSA, and testosterone data to meet the criteria of mCRPC. Patients who had received either chemotherapy or radium-223 for mCRPC prior to the initiation of ARAT and patients who had received ARAT for the treatment of mHSPC were excluded. Due to the limited number of cases and challenges in grouping, we did not include patients (n = 11) who received a second-line ARAT after failure or intolerance of a first-line ARAT from the study.

Demographic, pathological, and clinical data, including other treatment modalities for prostate cancer (chemotherapy or radium-223), type of metastatic disease manifestations (recurrence after prior local therapy or initial metastatic cancer), disease volume (high or low), Eastern Cooperative Oncology Group (ECOG) performance status, PSA measurements, and the prescription of ARATs were collected. If patients did not have the abovementioned data valid and available, they were excluded. All patients were followed until death, last follow-up, or July 22, 2022 (data cutoff date), whichever occurred first.

Patients were divided into two groups based on the type of ARAT used, abiraterone acetate or enzalutamide. PSA nadir was defined as the lowest PSA level detected since the beginning of the initial ARAT followed by two consecutive increases in PSA. Time to PSA nadir (TTN) was defined as the duration from the onset of ARAT to the day when the PSA nadir was observed. Metastases were detected with computed tomography and whole-body bone scan prior to initiating ARAT. High-volume disease was defined as visceral metastases or at least 4 bone metastases, including 1 or more metastases out of axis. Other states were defined as low-volume disease [17].

### Study endpoints

The primary endpoint of the study was OS defined as the interval from the start of ARAT to the last follow-up or death. The secondary endpoints were the percentage of PSA decline, PSA nadir, and time to PSA nadir (TTN). All parameters were calculated from the start of ARAT to the event of interest. The factors tested for the association with OS included ECOG performance status, initial stage, grade group, local treatment, disease volume, type of ARAT, PSA nadir, TTN, and PSA 90% decline (defined as whether PSA reached a 90% decline).

### Statistical analysis

Categorical variables were reported as counts and percentages; continuous variables were reported as medians and interquartile ranges (IQR). Comparisons were made using the chi-square test for categorical variables and an independent t-test for continuous variables. Kaplan–Meier survival analyses were applied for depicting OS. Inversed probability of treatment weighing-adjusted Cox proportional hazards model was performed to determine the association between various factors and OS. The optimal cutoff values for PSA nadir and TTN were calculated using receiver operating characteristic (ROC) curve analysis. All statistical analyses were performed using commercial statistical software SPSS (version 25.0; IBM Corp, SPSS, Inc, Chicago, IL, USA). Summary statistics were presented with Excel (Microsoft Office Professional Plus 2019) and PRISM program (GraphPad, V8.0.1).

## Results

A total of 202 patients with mCRPC were included and analyzed with valid information during the study period. The median follow-up period after cancer diagnosis and follow-up period after initiation of ARAT were 6.9 years (IQR 4.3–9.6) and 25.1 months (IQR 17.7–35.7), respectively. The percentage of patients lost to follow-up was 4.5%. The median age at the start of ARAT was 77.9 years old (IQR 70.7–83.6). Patients were categorized into abiraterone and enzalutamide groups according to the type of ARAT received for first-line treatment for mCRPC (Table 1). The baseline demographics and clinical characteristics at diagnosis were similar between the two groups, including ECOG performance status, the manifestation of the disease (localized or metastatic), grade group, comorbidity, and tumor volume based on CHAARTED criteria [17] assessed at the development of metastatic disease. There were no statistically significant differences between the two groups in terms of age at the time of ARAT treatment, PSA values at the start of ARAT treatment, the number of subsequent chemotherapy treatments, and the use of radium223 as second- or later-line therapy.

### Treatment response of ARAT

**Fig. 1:**
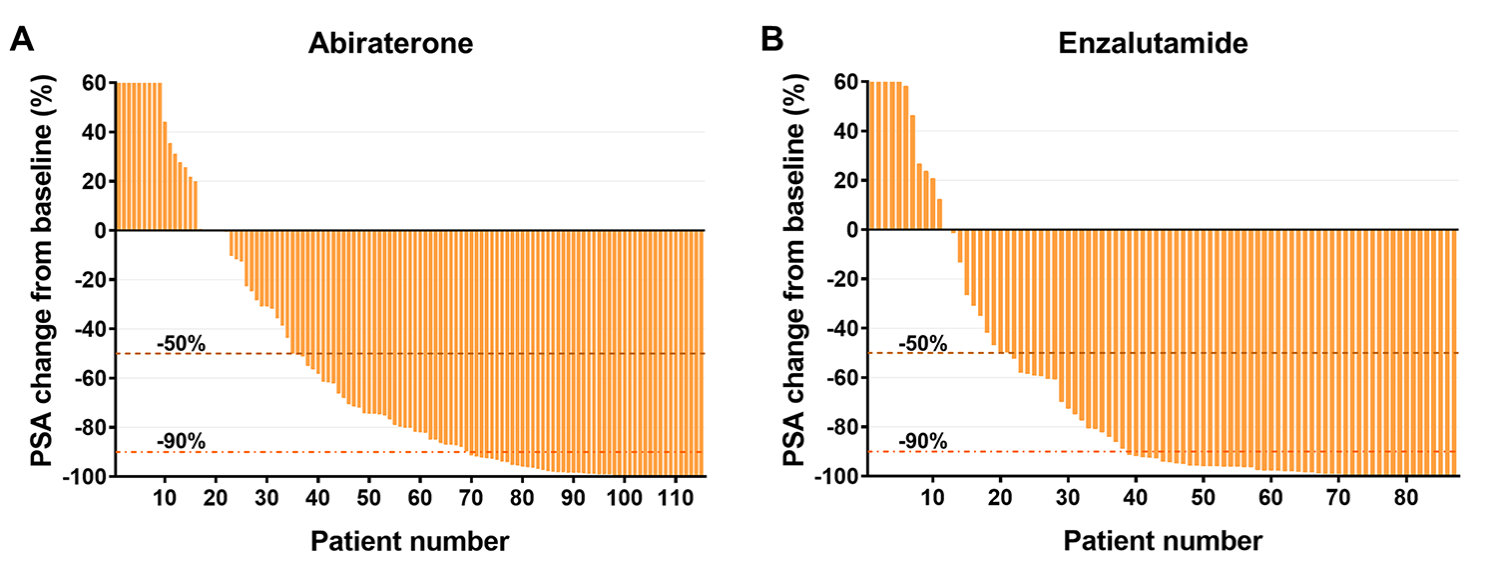
**Waterfall plots of maximum prostate-specific antigen (PSA) change from baseline in patients with mCRPC treated with novel androgen receptor axis-targeted therapies (ARAT).** Patients treated with (A) abiraterone acetate or (B) enzalutamide

PSA nadir (p = 0.083) and the percentage of patients with a PSA nadir > 2 (p = 0.084) did not differ significantly between the two groups (Table 1). The persistence of PSA decline, as assessed by TTN, the enzalutamide group was able to continue effective PSA decline for a longer time than the abiraterone group (5.5 months versus 4.7 months, p = 0.019). When evaluating the response to PSA reduction following ARAT treatment, there was no significant difference (p = 0.391) in the percentage to which the two groups were able to achieve a > 50% reduction from baseline. The enzalutamide group showed a higher percentage of PSA decline > 90% from baseline compared to the abiraterone group, with a rate of 56% versus 40% in the abiraterone group (p = 0.021). Waterfall plots of change from baseline PSA for each patient are shown in Fig. 1.

**Table 1:** Background of patients with mCRPC treated with first-line novel androgen receptor axis-targeted therapies (ARAT).

	Abiraterone	Enzalutamide	p-value
	**n = 115**	**n = 87**	
	**median (IQR)/ number (%)**		
Demographics at Diagnosis			
ECOG PS > 1	8 (7%)	5 (6%)	0.729
Initial Stage			0.5
Localized	24 (21%)	22 (25%)	
Metastatic	91 (79%)	65 (75%)	
Grade Group			0.76
1–3	35 (30%)	29 (33%)	
4–5	80 (70%)	58 (67%)	
Local Treatment			0.458
No	25 (22%)	22 (25%)	
Yes	90 (78%)	65 (75%)	
Comorbidity			
Hypertension	33 (29%)	24 (28%)	0.876
Diabetes Mellitus	15 (13%)	13 (15%)	0.837
Coronary Artery Disease	13 (11%)	9 (10%)	1
CHAARTED criteria			0.146
Low Volume	35 (30%)	18 (21%)	
High Volume	80 (70%)	69 (79%)	
Treatment information			
Age at ARAT Treatment	78.3 (69.8–83.6)	77.2 (71.8–83.9)	0.994
PSA at Start of ARAT	18.2 (4.8–78.8)	10.1 (4.0-33.5)	0.084
Median time on ARAT, months	24.5 (17.6–34.6)	26.3 (17.8–39.1)	0.185
Patients still on ARAT at EOS	20 (17.4%)	29 (33%)	0.009
No. of cycles of DTX			0.589
0	95 (82%)	69 (79%)	
1–5	10 (9%)	8 (9%)	
>=6	10 (9%)	10 (12%)	
Radium223	21 (18%)	25 (29%)	0.079
Outcome Parameters			
PSA nadir	2.46 (0.21–23.5)	0.75 (0.12–10.21)	0.083
PSA nadir > 2 ng/mL	59 (51%)	34 (39%)	0.084
Time to PSA nadir (months)	4.7 (1.9–8.3)	5.5 (2.7–11.0)	0.019
Time to PSA nadir > = 7 months	34 (30%)	38 (44%)	0.038
PSA decline > 50%	81 (70%)	66 (76%)	0.391
PSA decline > = 90%	46 (40%)	49 (56%)	0.021

mCRPC, metastatic castration-resistant prostate cancer; IQR, interquartile range; ECOG PS, Eastern Cooperative Oncology Group performance status; ARAT, androgen receptor axis-targeted therapies; EOS, end of study; DTX, docetaxel

### Survival outcomes of ARAT

**Fig. 2:**
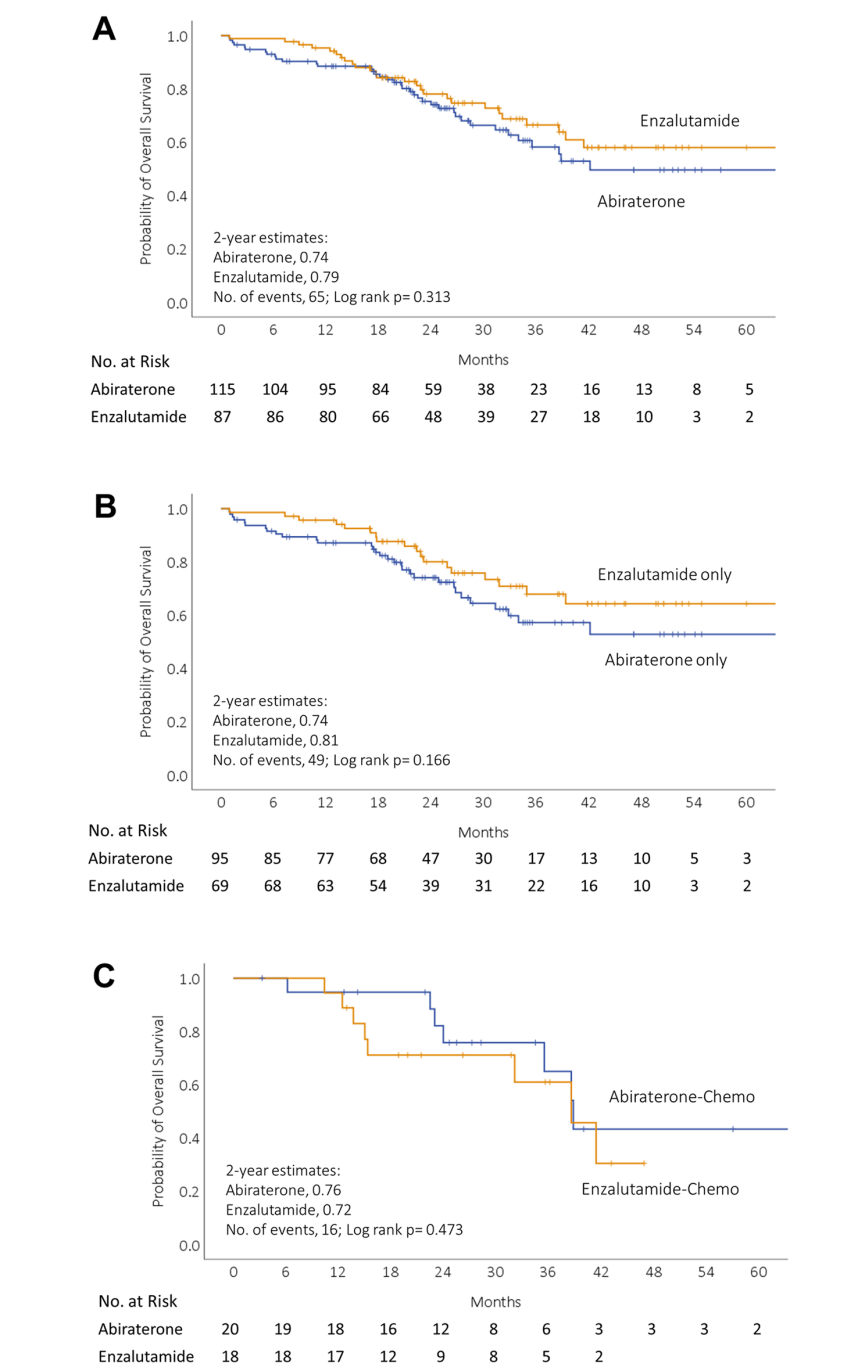
**Kaplan–Meier curves for overall survival from the initiation of first-line novel androgen receptor axis-targeted therapies (ARAT) for patients with mCRPC.** Kaplan–Meier curves for overall survival (A) in overall population treated with abiraterone acetate (blue) or enzalutamide (yellow); (B) in subsets of patients treated with the first-line ARAT agents without a second-line treatment, abiraterone only (blue) or enzalutamide only (yellow). (C) in subsets of patients treated with the first-line ARAT agents followed by chemotherapy, abiraterone-chemo (blue) or enzalutamide-chemo (yellow)

There was no statistically significant difference in OS observed between first-line treatment with abiraterone and enzalutamide, as evidenced by 2-year survival rates of 74% and 79%, respectively (p = 0.313) (Fig. 2A). The median OS for patients receiving first-line abiraterone was 42.1 months (IQR 20.6–63.7) and the median OS for those receiving first-line enzalutamide had not yet been reached. We divided patients into ARAT only or ARAT with subsequent chemotherapy as subgroup analysis. Patient demographic data were categorized into subgroups based on the type of ARAT they received, and whether or not they subsequently received chemotherapy (**Supplementary Table 1**). Patients that received subsequent chemotherapy were significantly younger than those who received an ARAT alone. There was no difference in ECOG PS or prevalence of cardiovascular comorbidities between the two groups. In the ARAT-alone group, there was a trend towards longer OS for those who received enzalutamide compared to abiraterone, though it did not reach statistical significance (Fig. 2B).” Similarly, if chemotherapy was used after ARAT, there was no significant difference in survival between the two groups (Fig. 2C).

The median survival of mCRPC patients treated with first-line ARAT followed by chemotherapy after failure from ARAT was 38.8 months (95% CI 34.9–42.7). The median survival of patients with ARAT only is not yet reached.

### Cutoff values for patient stratification

**Fig. 3:**
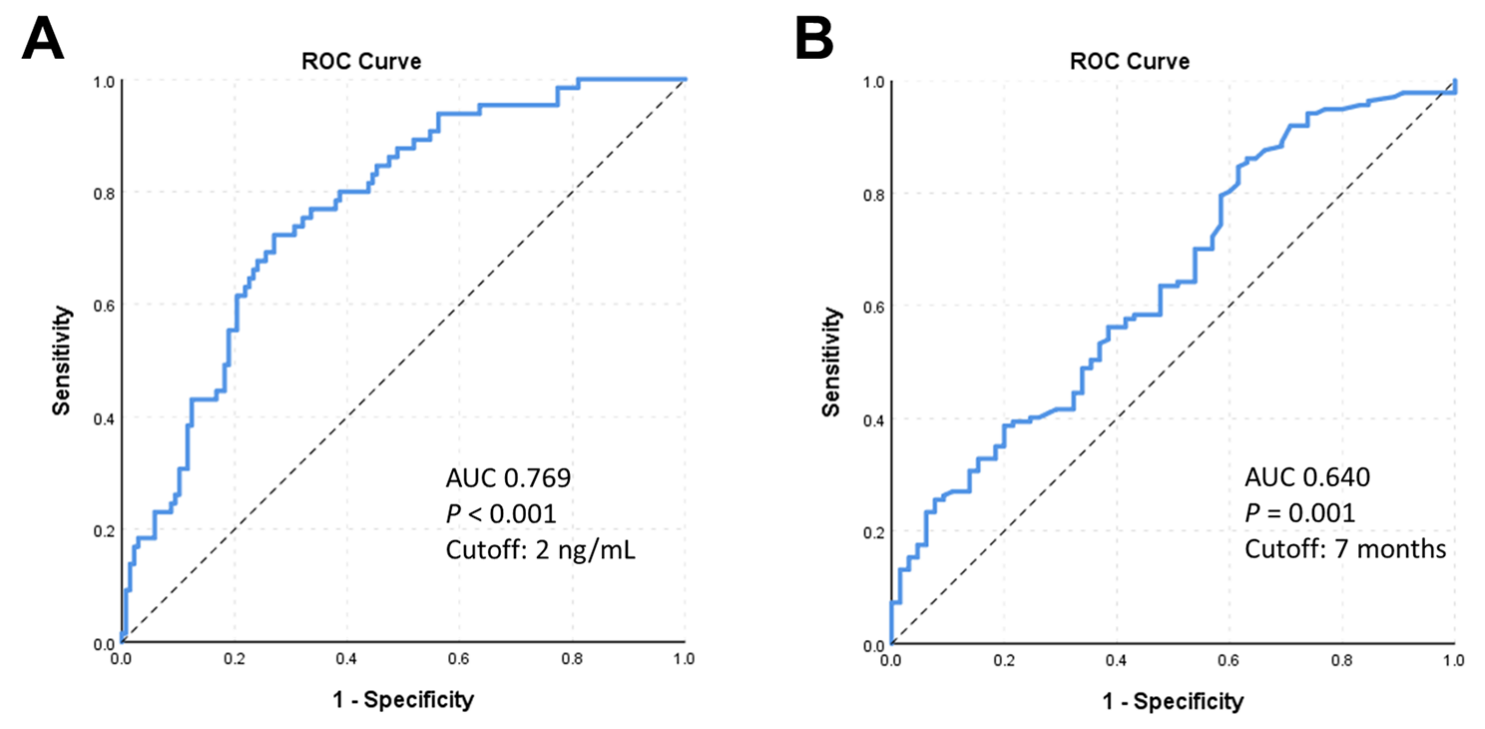
**ROC curves analysis on overall survival from the initiation of first-line novel androgen receptor axis-targeted therapies (ARAT).** ROC curves for (A) PSA nadir and (B) time to PSA nadir on overall survival

We applied ROC curve analyses to determine optimal cutoff values for PSA nadir (Fig. 3A) and TTN (Fig. 3B) on OS. Higher PSA nadir after ARAT treatment was associated with higher mortality as shown with an area under the curve (AUC) of 0.769 (P < 0.001) indicating a cutoff PSA of 2 ng/mL. Shorter TTN after ARAT indicated faster treatment failure and was associated with lower OS, as shown with an area under the curve (AUC) of 0.640 (p = 0.001) indicating a cutoff TTN of 7 months. Based on these two cutoff values, patients were grouped for comparison and subsequent multivariable analysis.

### Factors associated with OS in mCRPC receiving first-line ARAT

In univariate analysis with the Cox proportional hazards model, clinical indicators at the time of diagnosis, including initial stage, tumor grade groups, local treatment or not, and disease volume, did not show a significant association with predicting the OS following first-line ARAT treatment (Table 2). TTN < 7 months, PSA nadir > 2 ng/mL, and failure to achieve a PSA decline ≧ 90% from baseline were associated with worse OS. Due to the considerable overlap between PSA nadir > 2 ng/mL and PSA decline < 90%, we chose to compare them separately in the multivariate models. Multivariate model 1 revealed that PSA nadir > 2 ng/mL [hazard ratio (HR) 7.04, 95% CI 3.74–13.25, p < 0.001] and TTN < 7 months (HR 2.18, 95% CI 1.19–3.99, p = 0.012) were factors independently associated with shorter OS. Multivariate model 2 similarly revealed that patients with PSA decline < 90% or TTN < 7 months were associated with worse OS.

**Table 2:** Univariable and multivariate analysis with Cox proportional hazards model for overall survival in patients with mCRPC treated with first-line novel androgen receptor axis-targeted therapies (ARAT).

		Univariate			Multivariate Model 1			Multivariate Model 2		
Variables	**Prognostic factors**	**HR**	**95% CI**	**p value**	**HR**	**95% CI**	**p value**	**HR**	**95% CI**	**p value**
Age at ARAT	-	1.03	0.99–1.06	0.069	1.03	0.99–1.06	0.07	1.03	1.00-1.07	0.037
ECOG PS	> 1	2.24	1.02–4.93	0.044	1.42	0.63–3.18	0.399	2.14	0.95–4.81	0.065
Initial Stage	Metastatic	0.97	0.53–1.78	0.913	-	-	-	-	-	-
Grade Group	4–5	1.24	0.72–2.14	0.441	-	-	-	-	-	-
Local Treatment	No	1.84	0.84–4.03	0.1	-	-	-	-	-	-
CHAARTED	High-volume	1.13	0.62–2.04	0.693	-	-	-	-	-	-
Treatment	Abiraterone	1.29	0.79–2.13	0.314	-	-	-	-	-	-
Time to PSA nadir	< 7 months	2.97	1.68–5.24	< 0.001	2.18	1.19–3.99	0.012	2.4	1.33–4.33	0.004
PSA Nadir	> 2 ng/mL	8.95	4.84–16.56	< 0.001	7.04	3.74–13.25	< 0.001	-	-	-
PSA 90% Decline	< 90%	4.23	2.38–7.51	< 0.001	-	-	-	3.24	1.79–5.86	< 0.001

mCRPC, metastatic castration-resistant prostate cancer; HR, hazard ratio; CI, confidence interval; ARAT, androgen receptor axis-targeted therapies; ECOG PS, Eastern Cooperative Oncology Group performance status

### Subgroup analysis of patients with 2 prognostic factors

Therefore, TTN < 7 months and PSA nadir > 2 ng/mL were determined as poor prognostic factors based on the aforementioned analysis. Patients were stratified according to whether they had 0, 1, or 2 of these poor prognostic factors. In IPTW-adjusted multivariate model, patients with 2 poor prognostic factors had a worse OS (HR 9.21, 95% CI 5.14–16.49, p < 0.001) compared with patients with 0–1 poor prognostic factors (Table 3). Taking patients with 0 prognostic factors as a reference, patients with 1 prognostic factor and patients with 2 prognostic factors had 2.85- and 13.55-fold risk of death, respectively.

**Table 3:** IPTW-adjusted multivariate Cox proportional hazards model analysis in patients with two poor prognostic factors for OS from the initiation of androgen receptor-targeted agents (ARAT) as first-line treatment in patients with mCRPC

	Variables	Reference	HR	95% CI	p value
Model 1	2 factors	0–1 factor	9.21	5.14–16.49	< 0.001
Model 2	1 factor	0 factor	2.85	1.21–6.71	0.017
	2 factors	0 factor	13.55	5.96–30.82	< 0.001

Poor prognostic factor: Time to PSA nadir < 7months or PSA Nadir > 2 ng/mL

Adjusted variables: Age at ARAT Treatment, ECOG PS, Initial Stage, Grade Group, Local Treatment, and CHAARTED Criteria

IPTW, inverse probability of treatment weighting

**Fig. 4:**
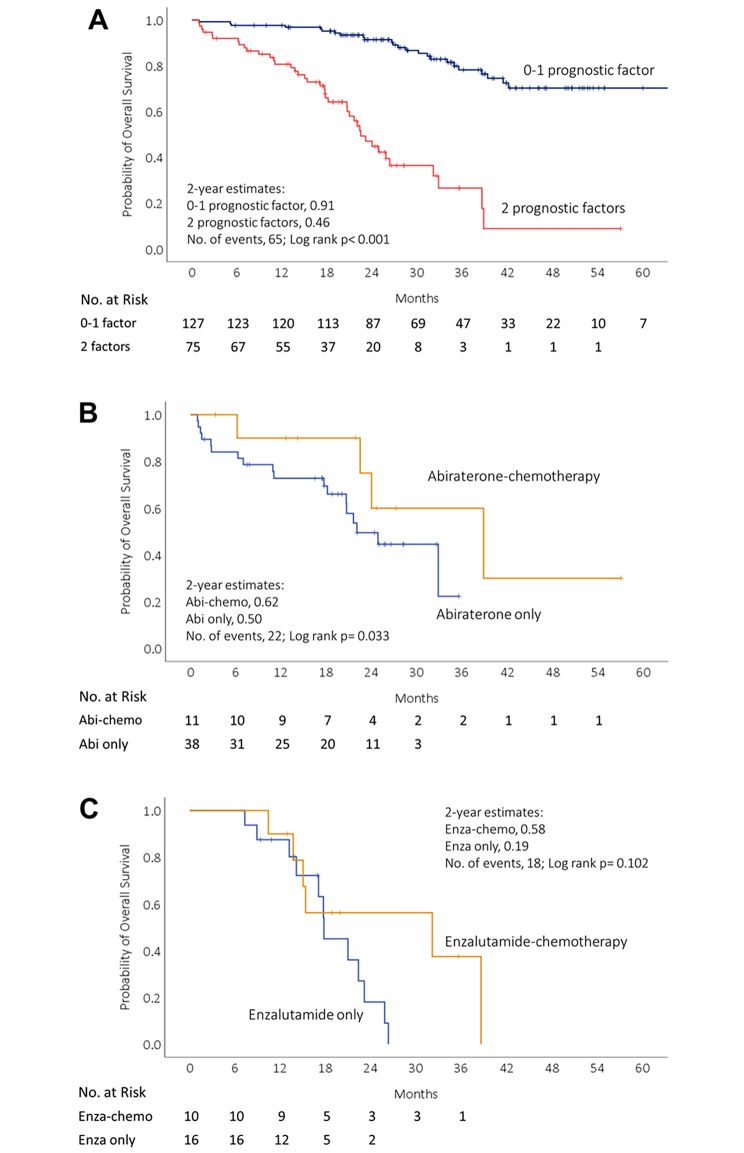
**Kaplan?Meier curves for overall survival from the initiation of first-line novel androgen receptor axis-targeted therapies (ARAT) in all patients and in subsets of patients with 2 poor prognostic factors based on subsequent therapy.** Kaplan?Meier curves for overall survival (A) among patients divided into two groups according to the number of poor prognostic factors, 0-1 factor (blue) or 2 factors (red). Kaplan?Meier curves for overall survival in subsets of patients with 2 poor prognostic factors (B) who remained on abiraterone only (blue) or who had abiraterone followed by chemotherapy (yellow); (C) who remained on enzalutamide only (blue) or who had enzalutamide followed by chemotherapy (yellow)

Based on the number of prognostic factors, patients were divided into two groups by 0–1 prognostic factors and 2 prognostic factors. The survival curves of the two subgroups were significantly different (Fig. 4A). The 2-year survival estimates were 91% with 0–1 prognostic factor group and 46% with 2 prognostic factors. We performed additional analyses on patients with 2 poor prognostic factors, stratified by the type of ARAT they received and subsequent receipt of chemotherapy. The patient demographics for these subgroups are presented in **Supplementary Table 2**. Patients who received subsequent chemotherapy were significantly younger. Patients who received abiraterone-chemotherapy demonstrated significantly longer OS compared to those who received abiraterone alone (p = 0.033) (Fig. 4B). The overall survival of patients who received enzalutamide followed by chemotherapy did not show a significant difference when compared to those who received enzalutamide alone (p = 0.102) (Fig. 4C).

## Discussion

Our retrospective cohort study examined the outcomes of mCRPC patients receiving first-line ARAT, specifically patient survival and PSA treatment response. While the enzalutamide group showed a higher rate of PSA decline (≥ 90% from baseline) compared to the abiraterone group, there was no significant difference in OS between the two groups. These real-world outcomes align with a meta-analysis of published phase III randomized trials in patients with mCRPC in both pre- and post-docetaxel settings demonstrating enzalutamide having a longer time to PSA progression and higher response rate compared to abiraterone, but no difference in OS [10]. Evidence suggests that enzalutamide shows better results than abiraterone with prednisone in terms of PSA progression and response rate but not OS.

In this present study, the median OS for patients who received first-line abiraterone was 42.1 months (IQR 20.6–63.7), which was longer than the OS reported in clinical trials involving first-line treatment with ARAT, 35.5 months with enzalutamide in PREVAIL trial [5] and 34.7 months in COU-AA-302 trial [4]. In the Prostate Cancer Registry data from 16 countries, the median OS for real-world first-line treatment of ARAT was even shorter at 27.1 months [18]. Our cohort’s longer OS may be due to ethnic differences in response to androgen-based treatments for prostate cancer. Prior studies have shown that Asian populations demonstrate greater effectiveness and longer survival times with ADT compared to other ethnic groups [19]. This result could potentially apply to the treatment outcomes with ARAT in mCRPC patients as well and warrants further investigation.

In this study, it was observed that 65% (131/202) patients received only one-line therapy for mCRPC, which is more than the clinical practice reflected in real-world data from the United States, where 51% of patients received only one life-prolonging therapy [20]. One of the primary reasons for this difference is that the effects of ADT and ARAT can persist for a prolonged period, especially among the Taiwanese population as mentioned previously, and Taiwan’s National Health Insurance provides continued reimbursement for these treatments. Furthermore, patients receiving ARAT alone had a higher median age compared to those receiving ARAT-chemotherapy, in both abiraterone and enzalutamide groups (Supplementary Table 1) as well as in patients with 2 poor prognostic factors (Supplementary Table 2). Moreover, patients may be reluctant to undergo chemotherapy due to its side effects and the psychological stress associated with the treatment. This reluctance may be even more pronounced among patients who have already received three to four years of treatment, as aging and physical decline become significant considerations when deciding on next-line therapy. Finally, it should be noted that 24% (49/202) of patients in this study were still using ARAT with an effective response at the time of analysis, indicating that the observation time for this cohort may not have been long enough.

We classified patients who had 2 poor prognostic factors, PSA nadir > 2 ng/mL and TTN < 7 months, as high risk for all-cause mortality. In the subgroup of patients with 2 poor prognostic factors who received abiraterone as first-line ARAT, the overall survival was longer for those who received subsequent chemotherapy. This suggests that subsequent chemotherapy may compensate for the less effective treatment of abiraterone. However, it was important to acknowledge that the patients who received subsequent chemotherapy were substantially younger than those who received an ARAT alone (67 v. 83 years) which may explain the prolonged OS in the subsequent chemotherapy groups, although there were no differences between the two groups in terms of ECOG PS and prevalence of cardiovascular comorbidities. In addition, our study had a relatively small sample size. Thus, our results should be considered hypothesis-generating and require further validation in larger studies. Based on these findings, providers may consider a switch to chemotherapy as soon as possible if a patient is found to have a poor response after receiving abiraterone as first-line treatment for mCRPC, particularly in those with 2 poor prognostic factors. Compared to abiraterone, patients treated with enzalutamide as first-line ARAT showed a more favorable response, resulting in a lower need for second-line therapy due to treatment failure. More importantly, if patients could continue to use ARAT, it indicated that the treatment effect was good. In contrast, patients who required a switch to chemotherapy were associated with a more severe disease pattern and poorer outcome. Consequently, the survival benefit of using chemotherapy in the enzalutamide cohort was challenging to discern.

Past studies have examined the predictive effect of PSA nadir and TTN on disease progression in metastatic prostate cancer patients treated with ADT. PSA nadir higher than 0.2 ng/ml and time to PSA nadir less than 10 months were predictors of disease progression and refractory disease in metastatic prostate cancer [15, 21]. In patients with CRPC, studies also showed the impact of PSA nadir and TTN after ADT treatment on the OS [16] and cancer-specific survival [22]. In terms of TTN, 7 months was reportedly the cut-off time interval for the evaluation of effective treatment, especially the efficacy of conventional ADT in metastatic prostate cancer [23]. This study is the first to evaluate a TTN cut-off point in patients with mCRPC treated with first-line ARAT with a TTN of 7 months also being supported by our ROC curve analysis. In terms of PSA nadir, our ROC curve analysis demonstrated a PSA of 2 ng/mL as a significant cut-off value for stratification.

This study has a few limitations. This was a retrospective analysis and the sample size was relatively small. There might be selection bias or other confounding factors, even though we used adjusted multivariate and IPTW model to reduce the interference of these factors when analyzing prognostic factors. Furthermore, since this study used Asian ethnic groups, the proposed cut-off points need to be carefully interpreted if they are to be applied to other ethnic groups. Therefore, our proposed prognostic factors still need to be validated with other real-world data such as PREMISE, a large European observational study in mCRPC patients treated with enzalutamide [24]. OS data from PREMISE trial has not been published yet. In addition, due to the excellent effect of ARAT and long survival period of the patients in this study, some patients still continue to receive ARAT. The median survival of patients receiving first-line ARAT only is premature. To our knowledge, this study is the first to investigate PSA nadir and TTN as predictors of ARAT effectiveness as first-line treatment in patients with mCRPC. These two well-defined prognostic factors with clear cut-off points can be easily used by clinicians as a reference for evaluating treatment effects and anticipating patient outcomes.

## Conclusions

In conclusion, there was no significant OS difference between abiraterone and enzalutamide in the choice of first-line ARAT for mCRPC patients. These two prognostic factors, PSA nadir > 2 ng/mL and TTN < 7 months after first-line ARAT, were significantly associated with worse survival in patients with mCRPC.

## Electronic supplementary material

Below is the link to the electronic supplementary material.

Supplementary Material 1

## Data Availability

The datasets used and/or analyzed during the current study available from the corresponding author on reasonable request.

## Abbreviations

mCRPC: metastatic castration-resistant prostate cancer
ARATs: androgen receptor axis-targeted therapies
OS: overall survival
TTN: time to PSA nadir
PSA: prostate-specific antigen
ECOG: Eastern Cooperative Oncology Group
IQR: interquartile range
ROC: receiver operating characteristic
HR: hazard ratio
