# Supplementary material for: Survival outcomes and prognostic factors for first-line abiraterone acetate or enzalutamide in patients with metastatic castration-resistant prostate cancer
Source: BMC Cancer. 2023 Jun 20;23:568. doi: 10.1186/s12885-023-10885-4 (PMC10283214; doi:10.1186/s12885-023-10885-4)
Supplement: Supplementary file 1 — Supplementary Material 1 [file 12885_2023_10885_MOESM1_ESM.docx]

|  | Abiraterone (n=115) | | | Enzalutamide (n=87) | |  |
| --- | --- | --- | --- | --- | --- | --- |
|  | **Abiraterone only** | **Abiraterone-Docetaxel** | **p-value** | **Enzalutamide only** | **Enzalutamide-Docetaxel** | **p-value** |
|  | n=95 | n=20 |  | n=69 | n=18 |  |
|  | median (IQR)/number (%) | |  | median (IQR)/number (%) | |  |
| Demographics at Diagnosis | | | | | | |
| ECOG PS>1 | 7 (7%) | 1 (5%) | 0.705 | 2 (3%) | 3 (17%) | 0.058 |
| Initial Stage |  |  | 0.477 |  |  | 0.737 |
| Localized | 21 (22%) | 3 (15%) |  | 18 (26%) | 4 (22%) |  |
| Metastatic | 74 (78%) | 17 (85%) |  | 51 (74%) | 14 (78%) |  |
| Grade Group |  |  | 0.099 |  |  | 0.261 |
| 1-3 | 32 (34%) | 3 (15%) |  | 25 (36%) | 4 (22%) |  |
| 4-5 | 63 (66%) | 17 (85%) |  | 44 (64%) | 14 (78%) |  |
| Local Treatment |  |  | 0.477 |  |  | 0.737 |
| No | 21 (22%) | 4 (20%) |  | 18 (26%) | 4 (22%) |  |
| Yes | 74 (78%) | 16 (80%) |  | 51 (74%) | 14 (78%) |  |
| Comorbidity |  |  |  |  |  |  |
| Hypertension | 27 (28%) | 6 (30%) | 0.887 | 19 (28%) | 5 (28%) | 0.984 |
| Diabetes Mellitus | 12 (13%) | 3 (15%) | 0.775 | 10 (14%) | 3 (17%) | 0.818 |
| Coronary Artery Disease | 12 (13%) | 1 (5%) | 0.327 | 7 (10%) | 2 (11%) | 0.905 |
| CHAARTED criteria* |  |  | 0.029 |  |  | 0.26 |
| Low Volume | 33 (35%) | 2 (10%) |  | 16 (23%) | 2 (11%) |  |
| High Volume | 62 (65%) | 18 (90%) |  | 53 (77%) | 16 (89%) |  |
| Treatment information | | | | | | |
| Age at ARAT Treatment | 79.5 (72.6-84.7) | 71.63 (63.5-78.5) | 0.03 | 78.3 (73.1-84.9) | 74.1 (65.0-78.2) | 0.02 |
| PSA at Start of ARAT | 17.9 (3.4-62.1) | 27.3 (10.9-127.5) | 0.773 | 8.7 (3.2-28.3) | 16.7 (6.9-43.8) | 0.056 |
| No. of cycles of DOC |  |  |  |  |  |  |
| 0 | 95 (100%) | 0 (0%) |  | 69 (100%) | 0 (0%) |  |
| 1-5 | 0 (0%) | 10 (50%) |  | 0 (0%) | 8 (44%) |  |
| >=6 | 0 (0%) | 10 (50%) |  | 0 (0%) | 10 (56%) |  |
| Radium223 | 18 (19%) | 3 (15%) | 0.678 | 15 (22%) | 10 (56%) | 0.005 |
| Outcome Parameters | | | | | | |
| PSA nadir | 1.97 (0.12-19.4) | 7.47 (0.68-28.17) | 0.203 | 0.45 (0.1-8.5) | 2.9 (1.0-31.9) | 0.001 |
| PSA nadir >=2 | 46 (48%) | 13 (65%) | 0.178 | 24 (35%) | 10 (56%) | 0.108 |
| Time to PSA nadir (months) | 4.4 (1.8-7.9) | 6.1 (2.3-9.9) | 0.773 | 3.0 (0.8-5.1) | 7.5 (3.9-13.0) | 0.001 |
| Time to PSA nadir < 7 months | 26 (27%) | 8 (40%) | 0.261 | 36 (52%) | 2 (11%) | 0.002 |
| PSA decline > 50% | 66 (69%) | 15 (75%) | 0.623 | 52 (75%) | 14 (78%) | 0.831 |
| PSA decline > 90% | 40 (42%) | 6 (30%) | 0.315 | 43 (62%) | 6 (33%) | 0.027 |
| ARATs = androgen receptor axis-targeted therapies | | | | | | |
| *CHAARTED criteria was determined at the development of metastatic disease | | | | | | |

**Supplementary Table 1. Background of patients with mCRPC treated with first-line novel androgen receptor axis-targeted therapies (ARAT) by subgroups.**

|  | Abiraterone (n=49) | | | Enzalutamide (n=26) | | |
| --- | --- | --- | --- | --- | --- | --- |
|  | **Abiraterone only** | **Abiraterone-Docetaxel** | **p-value** | **Enzalutamide only** | **Enzalutamide-Docetaxel** | **p-value** |
|  | n=38 | n=11 |  | n=16 | n=10 |  |
|  | median (IQR)/number (%) | |  | median (IQR)/number (%) | |  |
| Demographics at Diagnosis | | | | | | |
| ECOG PS>1 | 5 (13%) | 0 (0%) | 0.204 | 1 (6%) | 2 (20%) | 0.286 |
| Initial Stage |  |  | 0.666 |  |  | 0.271 |
| Localized | 13 (34%) | 3 (27%) |  | 2 (12.5%) | 3 (30%) |  |
| Metastatic | 25 (66%) | 8 (73%) |  | 14 (87.5%) | 7 (70%) |  |
| Grade Group |  |  | 0.557 |  |  | 0.937 |
| 1-3 | 14 (37%) | 3 (27%) |  | 3 (19%) | 2 (20%) |  |
| 4-5 | 24 (63%) | 8 (73%) |  | 13 (81%) | 8 (80%) |  |
| Local Treatment |  |  | 0.521 |  |  | 0.271 |
| No | 5 (13%) | 3 (27%) |  | 2 (12.5%) | 3 (30%) |  |
| Yes | 33 (87%) | 8 (73%) |  | 14 (87.5%) | 7 (70%) |  |
| Comorbidity |  |  |  |  |  |  |
| Hypertension | 15 (39%) | 3 (27%) | 0.46 | 4 (25%) | 1 (10%) | 0.345 |
| Diabetes Mellitus | 7 (18%) | 3 (27%) | 0.521 | 4 (25%) | 1 (10%) | 0.345 |
| Coronary Artery Disease | 5 (13%) | 1 (9%) | 0.717 | 3 (19%) | 1 (10%) | 0.547 |
| CHAARTED criteria* |  |  | 0.017 |  |  | 0.086 |
| Low Volume | 14 (37%) | 0 (0%) |  | 4 (25%) | 0 (0%) |  |
| High Volume | 24 (63%) | 11 (100%) |  | 12 (75%) | 10 (100%) |  |
| Treatment information | | | | | | |
| Age at ARAT Treatment | 81.9 (76.3-85.4) | 67.4 (60.5-74.7) | 0.008 | 83.9 (77.3-87.4) | 67.4 (64.0-73.6) | 0.004 |
| PSA at Start of ARAT | 57.1 (12.8-131.7) | 62.8 (16.1-132.2) | 0.939 | 74.1 (14.3-248.4) | 13.7 (6.9-47.1) | 0.226 |
| No. of cycles of DOC |  |  | >1.0 |  |  | >1.0 |
| 0 | 38 (100%) | 0 (0%) |  | 16 (100%) | 0 (0%) |  |
| 1-5 | 0 (0%) | 5 (45%) |  | 0 (0%) | 5 (50%) |  |
| >=6 | 0 (0%) | 6 (55%) |  | 0 (0%) | 5 (50%) |  |
| Radium223 | 10 (26%) | 0 (0%) | 0.057 | 8 (50%) | 7 (70%) | 0.315 |
| Outcome Parameters | | | | | | |
| PSA nadir | 21.53 (6.6-91.3) | 19.8 (7.9-50.2) | 0.939 | 29.6 (8.4-94.6) | 20.1 (3.4-48.4) | 1 |
| PSA nadir >=2 | 38 (100%) | 11 (100%) | >1.0 | 16 (100%) | 10 (100%) | >1.0 |
| Time to PSA nadir (months) | 2.0 (0.8-3.5) | 3.2 (2.2-6.0) | 0.446 | 3.6 (1.8-5.4) | 1.2 (0.7-3.6) | 0.226 |
| Time to PSA nadir < 7 months | 38 (100%) | 11 (100%) | >1.0 | 16 (100%) | 10 (100%) | >1.0 |
| PSA decline > 50% | 13 (34%) | 6 (55%) | 0.223 | 6 (38%) | 6 (60%) | 0.263 |
| PSA decline > 90% | 5 (13%) | 2 (18%) | 0.675 | 2 (12.5%) | 0 (0%) | 0.245 |
| ARATs = androgen receptor axis-targeted therapies | | | | | | |
| *CHAARTED criteria was determined at the development of metastatic disease | | | | | | |

**Supplementary Table 2. Background of patients with mCRPC with 2** **poor prognostic factors treated with first-line novel androgen receptor axis-targeted therapies (ARAT) by subgroups.**
